# Highly stable and reusable immobilized formate dehydrogenases: Promising biocatalysts for in situ regeneration of NADH

**DOI:** 10.3762/bjoc.12.29

**Published:** 2016-02-12

**Authors:** Barış Binay, Dilek Alagöz, Deniz Yildirim, Ayhan Çelik, S Seyhan Tükel

**Affiliations:** 1 Istanbul AREL University, Faculty of Science and Letters, Department of Molecular Biology and Genetics, Tepekent, Büyükcekmece, Istanbul, Turkey; 2 University of Cukurova, Vocational School of Imamoglu, Adana, Turkey; 3 University of Cukurova, Vocational School of Ceyhan, Adana, Turkey; 4 Gebze Technical University, Department of Chemistry, Gebze, Kocaeli, Turkey; 5 University of Cukurova, Faculty of Arts and Sciences, Department of Chemistry, 01330, Adana, Turkey

**Keywords:** biocatalysis, *Candida methylica*, formate dehydrogenase, Immobead 150, regeneration of NADH, stabilization

## Abstract

This study aimed to prepare robust immobilized formate dehydrogenase (FDH) preparations which can be used as effective biocatalysts along with functional oxidoreductases, in which in situ regeneration of NADH is required. For this purpose, *Candida methylica* FDH was covalently immobilized onto Immobead 150 support (FDHI150), Immobead 150 support modified with ethylenediamine and then activated with glutaraldehyde (FDHIGLU), and Immobead 150 support functionalized with aldehyde groups (FDHIALD). The highest immobilization yield and activity yield were obtained as 90% and 132%, respectively when Immobead 150 functionalized with aldehyde groups was used as support. The half-life times (*t*_1/2_) of free FDH, FDHI150, FDHIGLU and FDHIALD were calculated as 10.6, 28.9, 22.4 and 38.5 h, respectively at 35 °C. FDHI150, FDHIGLU and FDHIALD retained 69, 38 and 51% of their initial activities, respectively after 10 reuses. The results show that the FDHI150, FDHIGLU and FDHIALD offer feasible potentials for in situ regeneration of NADH.

## Introduction

Dehydrogenases are one of the most promising enzymes in biocatalysis since these enzymes have a great potential in the enantioselective reduction of ketones [1–2] and/or carbon–carbon double bonds [3–4] to produce optically active compounds. However, most dehydrogenases use an expensive cofactor such as NAD(H) or NADP(H) [5]. Therefore, the regeneration of the cofactor is required to decrease operational costs. NAD^+^-dependent formate dehydrogenase (FDH, EC 1.2.1.2) catalyzes oxidation of formate to carbon dioxide (CO_2_) [6]. FDH is industrially used as coenzyme for the regeneration of NADH [7–8], as sensor for the determination of formic acid [9], and as catalyst for the production of methanol or formate from CO_2_ [10–11]. It was reported that FDH is a promising enzyme for the regeneration of NADH since the reaction product of FDH-catalyzed formate oxidation is CO_2_ which does not interfere with the purification of the final product [12–13]. However, free FDHs have low thermal stability [14] and lack of reusability, therefore, the immobilization of FDH has been of increasing interest in the recent years. For example, Netto et al. [15] immobilized FDH from *Candida boidinii* on three different magnetic supports and the results showed that conversion rates and recycling values were changed depending on the support used for immobilization. Bolivar et al. [16] used different strategies for the immobilization of FDH from *Candida boidinii* and reported that the stabilization factors were changed depending on the immobilization protocol. Kim et al. [17] immobilized FDH from *Candida boidinii* as cross-linked enzyme aggregate (CLEA) and demonstrated that the residual activity and thermal stability of CLEA were strictly dependent on the type of cross-linker.

Epoxy group containing supports are widely used in enzyme immobilization studies to obtain highly stable enzyme preparations by using multi-point attachment strategies [18–20]. The immobilization mechanism of enzymes is based on the hydrophobic adsorption of enzymes onto the supports and then the covalent immobilization of enzymes. Besides, these supports are easily modified to generate new groups for the immobilization of enzymes with different mechanism. This allows us the preparation of biocatalysts with different properties [21–23]. Glutaraldehyde-activated supports have been extensively used in enzyme immobilization studies for many years [24]. However, the exact structure of the groups formed by glutaraldehyde is still under discussion, a Schiff base reaction between the carbonyl group of glutaraldehyde and the terminal amino functional group could be expected [25–26].

*Candida methylica* FDH is a dimeric enzyme [27] and it may be easily inactivated by the dissociation of its subunits depending on reaction conditions. Hence, the use of a proper immobilization technique and support could stabilize its dimeric form. In this study, NAD^+^-dependent FDH from *Candida methylica* was covalently immobilized onto Immobead 150, an epoxy group containing commercial support, and Immobead 150 support modified with ethylenediamine and then activated with glutaraldehyde, and Immobead 150 support functionalized with aldehyde groups. The optimum conditions of free and immobilized FDH preparations were determined for formate oxidation. The thermal stability of free and immobilized FDH preparations was tested at 35 and 50 °C. The operational stability studies of the immobilized FDHs were performed in a batch reactor. As far as we know, this is the first report regarding the covalent immobilization of *Candida methylica* FDH.

## Results and Discussion

**Figure 1:**
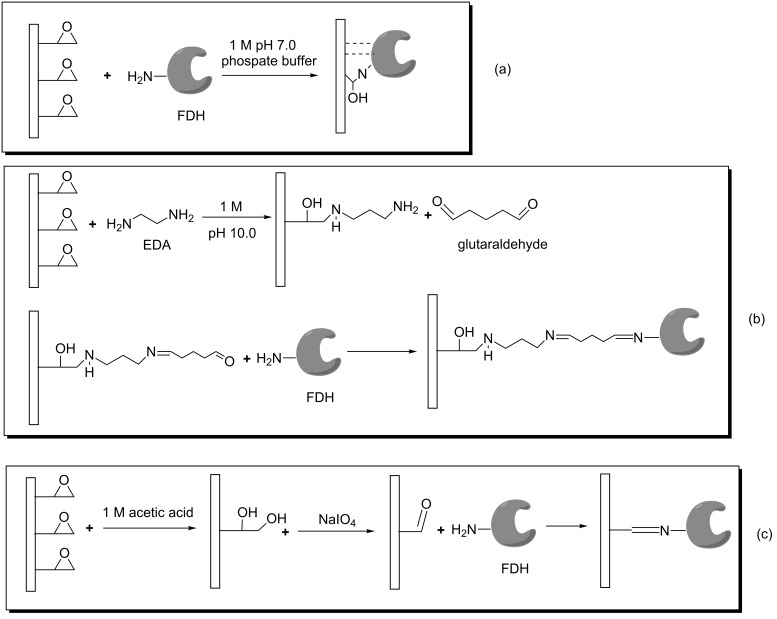
The immobilization scheme of FDH onto Immobead 150 and modified Immobead 150 supports.

It is well documented that one of the factors affecting the performance of an immobilized enzyme is the type of binding groups on the support which provides higher loading of enzyme and higher retention of activity [28]. Epoxy group containing supports are widely used in the immobilization of many enzymes through multi-point covalent attachments since epoxy groups can easily react with different nucleophiles highly abundant in the protein surface such as primary amine, sulfhydryl and carboxylic groups [21]. In this study, Immobead 150 was used as epoxy group containing supports for the immobilization of *C. methylica* FDH (Figure 1a). The amount of bound protein was determined as 85% of the initial loading protein per gram of Immobead 150 support and the immobilized FDH (FDHI150) showed 31% activity of the free FDH upon immobilization. Another commonly used strategy to covalently immobilize enzyme is using a bifunctional reagent glutaraldehyde. A Schiff base is formed between the carbonyl group of glutaraldehyde and the amino functional groups of the enzyme [29]. In this study, Immobead 150 support was modified with ethylenediamine and then activated with glutaraldehyde for the covalent immobilization of *C. methylica* FDH (Figure 1b). The amount of bound protein was determined as 75% of the initial loading protein per gram of the support and the immobilized FDH (FDHIGLU) showed 105% activity of the free FDH upon immobilization. In recent years, using short spacer arm containing supports has become very popular in enzyme immobilization due to enhancement of the stability of the enzyme [30]. In this study, Immobead 150 support was kept in 1 M acetic acid solution to produce vicinal diols and then the formed diols were oxidatively cleaved with NaIO_4_ to produce aldehyde groups onto the support (Figure 1c). The amount of bound protein was determined as 90% of the initial loading protein per gram of the support and the immobilized FDH (FDHIALD) showed 132% activity of the free FDH upon immobilization. The higher retention activities of FDHIGLU and FDHIALD may be related to the prevention of subunit dissociation depending on the immobilization procedure.

**Figure 2:**
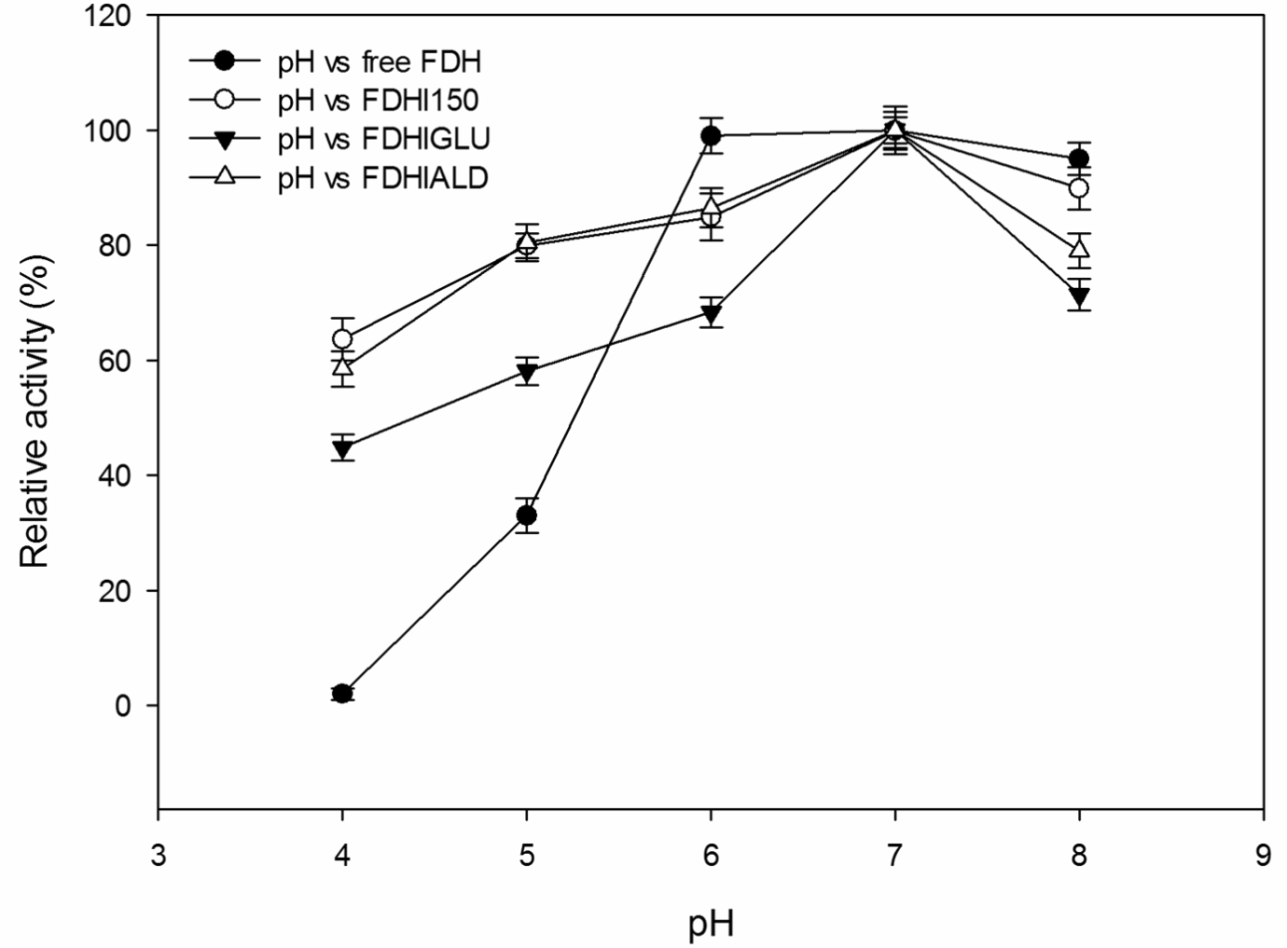
The effect of pH on the activities of free and immobilized FDH preparations. The FDH activity at pH 7.0 was taken as 100% for the each preparation. The experiments were run in triplicate.

The activity changes of free and immobilized FDH preparations depending on the medium pH were given in Figure 2. The free FDH showed 2% of its maximum activity at pH 4.0 whereas FDHI150, FDHIGLU and FDHIALD showed 64, 45 and 59% of their maximum activities at the same pH. The activities of both free and immobilized FDH preparations increased by increasing the pH and all the FDH preparations showed their maximum activities at pH 7.0. When the pH was further increased to 8.0, the determined activities of free FDH, FDHI150, FDHIGLU and FDHIALD were 95, 90, 71 and 79% of their maximum activities, respectively. Gao et al. [31] reported the optimal pH values were 7.0 for both free FDH and immobilized FDH onto polydopamine-coated iron oxide nanoparticles (PD-IONPs). The optimum pH values of the both free *Pseudomonas sp.* 101 FDH and its immobilized form onto glyoxylagarose were reported as 7.0 [16].

**Figure 3:**
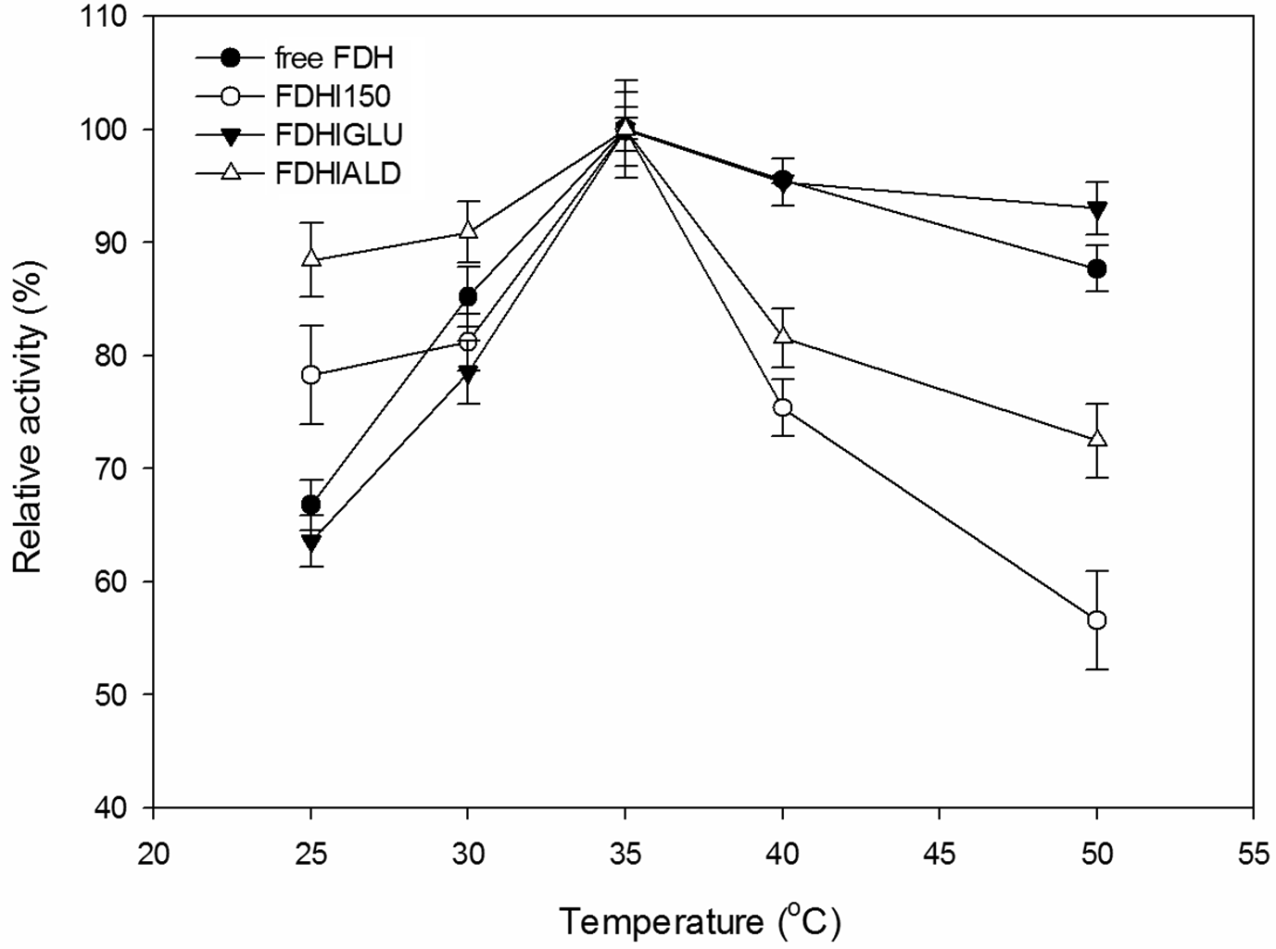
The effect of temperature on the activities of free and immobilized FDH preparations. The enzyme activity at 35 °C is taken as 100% for the each preparation. The experiments were run in triplicate.

The temperature–activity profiles of free and immobilized FDH preparations were given in Figure 3. The relative activities were 67, 78, 64 and 88%, respectively for free FDH, FDHI150, FDHIGLU and FDHIALD at 25 °C. The activities of free and immobilized FDHs increased with the temperature increasing from 25 to 35 °C and all the FDH preparations showed their maximum activities at 35 °C. The activities of free and immobilized FDH preparations decreased at the temperatures above 35 °C. Netto et al. [15] reported that the optimum temperature of free *Candida boidinii* FDH was 37 °C whereas the optimum temperatures of its immobilized forms were quite different depending on the used immobilization procedure. The optimum temperature of *C. boidinii* FDH immobilized onto magnetite nanoparticles silanized with (3-aminopropyl)triethoxysilane was 42 °C whereas the optimum temperature was 27 °C when this support was further coated with glyoxylagarose and then *C. boidinii* FDH was immobilized onto it.

**Figure 4:**
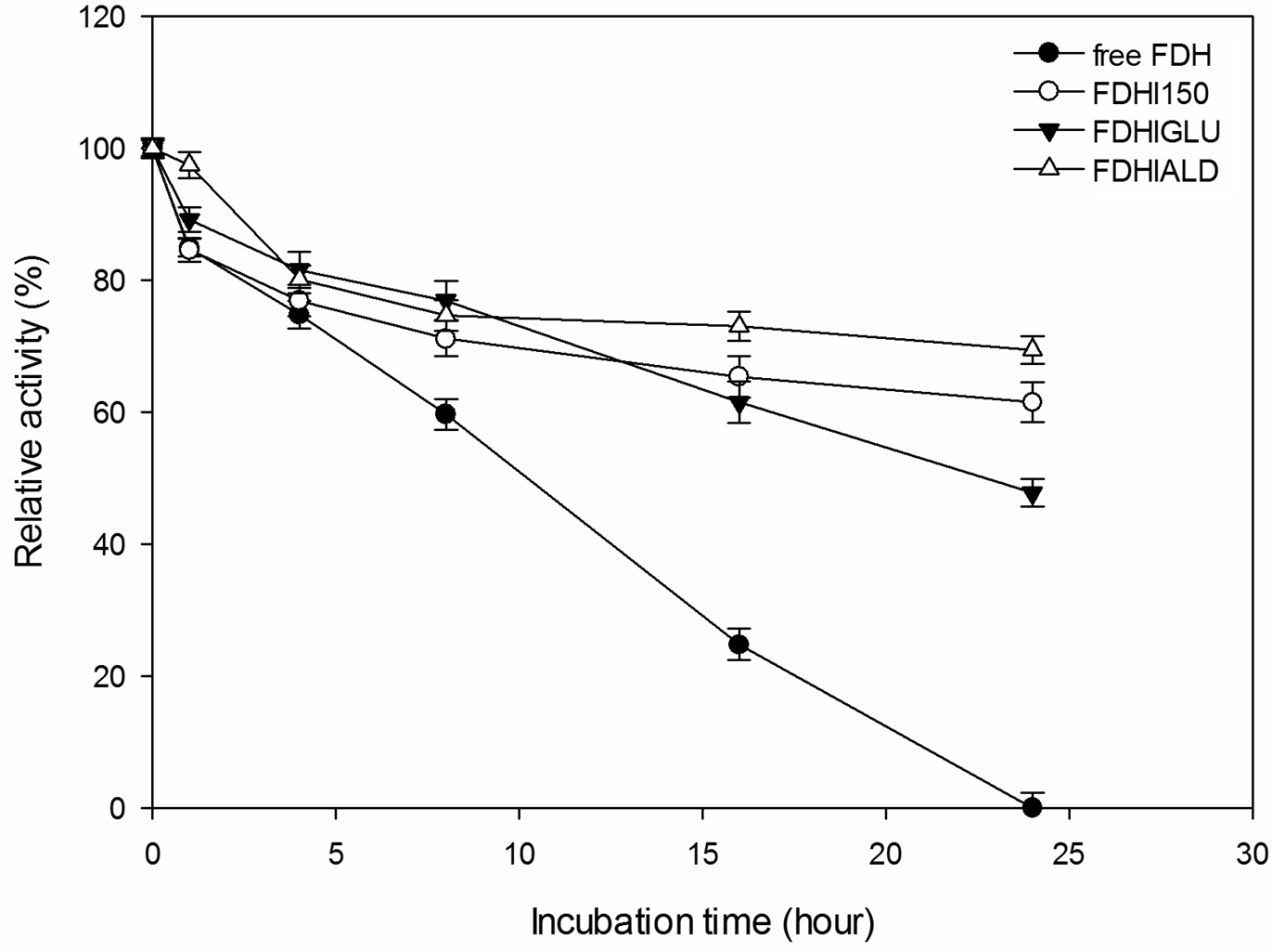
Thermal stability of free and immobilized FDH preparations at 35 °C.

**Figure 5:**
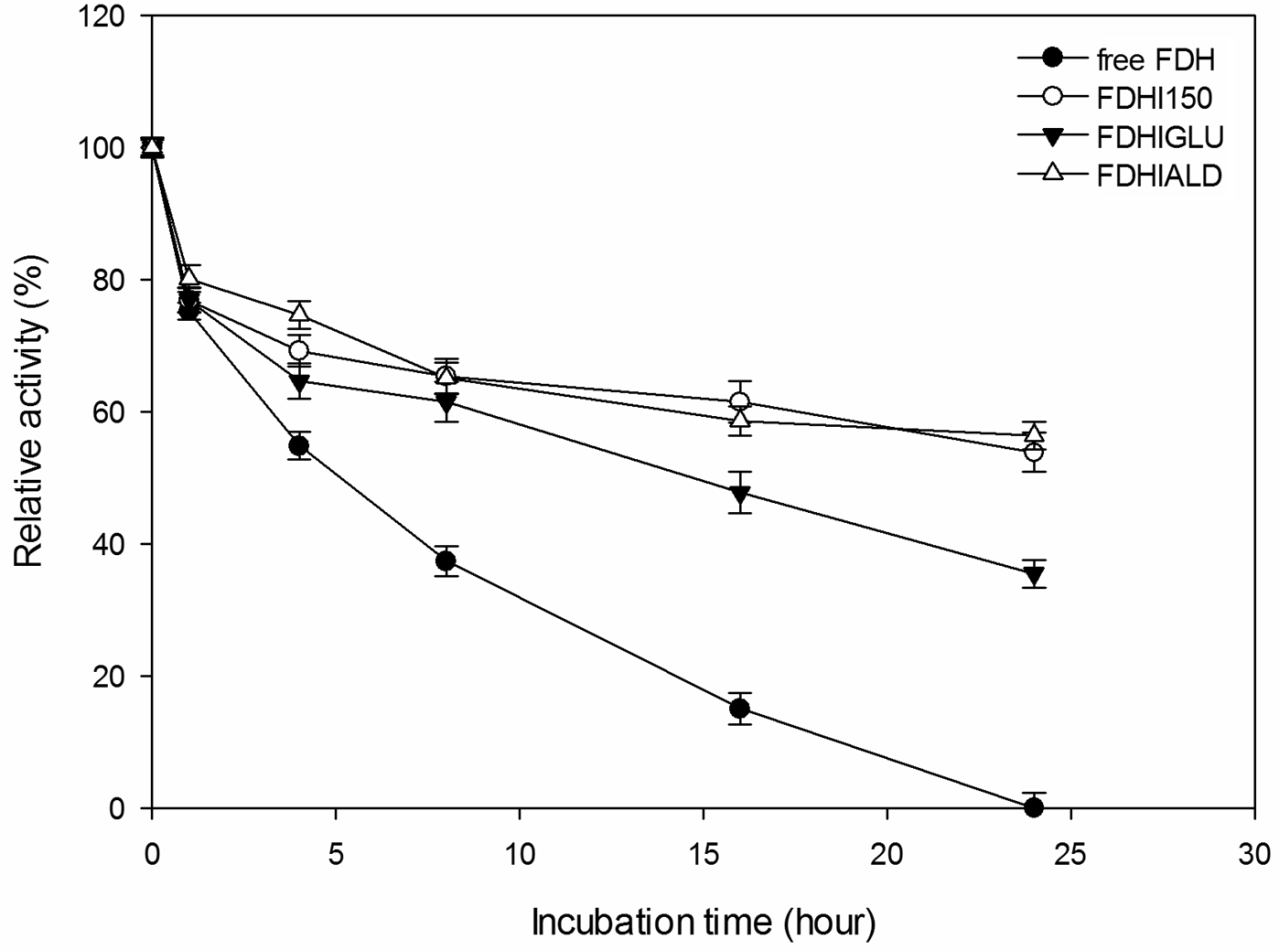
Thermal stability of free and immobilized FDH preparations at 50 °C.

It is generally expected from the covalently immobilized enzymes that they should be more durable against temperature inactivation than their free forms. As shown in Figure 4, the free FDH completely lost its initial activity at 35 **°**C after 24 h incubation time. However, FDHI150, FDHIGLU and FDHIALD retained 62, 48 and 69% of their initial activities, respectively at 35 °C after 24 h incubation time. At 50 °C, the free FDH completely lost its initial activity whereas FDHI150, FDHIGLU and FDHIALD retained 54, 35 and 56% of their initial activities, respectively after 24 h incubation time (Figure 5). The half-life times (*t*_1/2_) of free FDH, FDHI150, FDHIGLU and FDHIALD were calculated as 10.6, 28.9, 22.4 and 38.5 h, respectively at 35 °C (Table 1). The corresponding *t*_1/2_ values were 8.1, 23.1, 15.1 and 23.9 h at 50 °C. These results showed that the free FDH was stabilized 2.7, 2.1 and 3.1 fold at 35 °C and 2.8, 1.9 and 2.9 fold at 50 °C when it was immobilized onto Immobead 150, Immobead 150 via glutaraldehyde spacer arm, and Immobead 150 support functionalized with aldehyde group. These results show that a strong and stable imino bond could be formed between the aldehyde group of the modified Immobead 150 support and the terminal amino group of the enzyme at pH 6.0. Kim et al. [17] investigated the thermal stability of free *C. boidinii* FDH and immobilized FDH as cross-linked enzyme aggregates and reported that cross-linked enzyme aggregates of *C. boidinii* FDH prepared with dextrane polyaldehyde and glutaraldehyde showed 3.6 and 4.0 folds higher stability than the free FDH at 50 °C.

**Table 1:** The results of thermal stability experiments of free and immobilized FDH at 35 and 50 °C.

Catalyst	Temperature	*t_1/2_* (h)	*k_i_* (h^−1^)	Stabilization factor

Free FDH	35 °C	10.6	6.5 × 10^−2^	–
	50 °C	8.1	8.5 × 10^−2^	–

FDHI150	35 °C	28.9	2.4 × 10^−2^	2.7
	50 °C	23.1	3.0 × 10^−2^	2.8

FDHIGLU	35 °C	22.4	3.1 × 10^−2^	2.1
	50 °C	15.1	4.6 × 10^−2^	1.9

FDHIALD	35 °C	38.5	1.8 × 10^−2^	3.6
	50 °C	23.9	2.9 × 10^−2^	2.9

**Figure 6:**
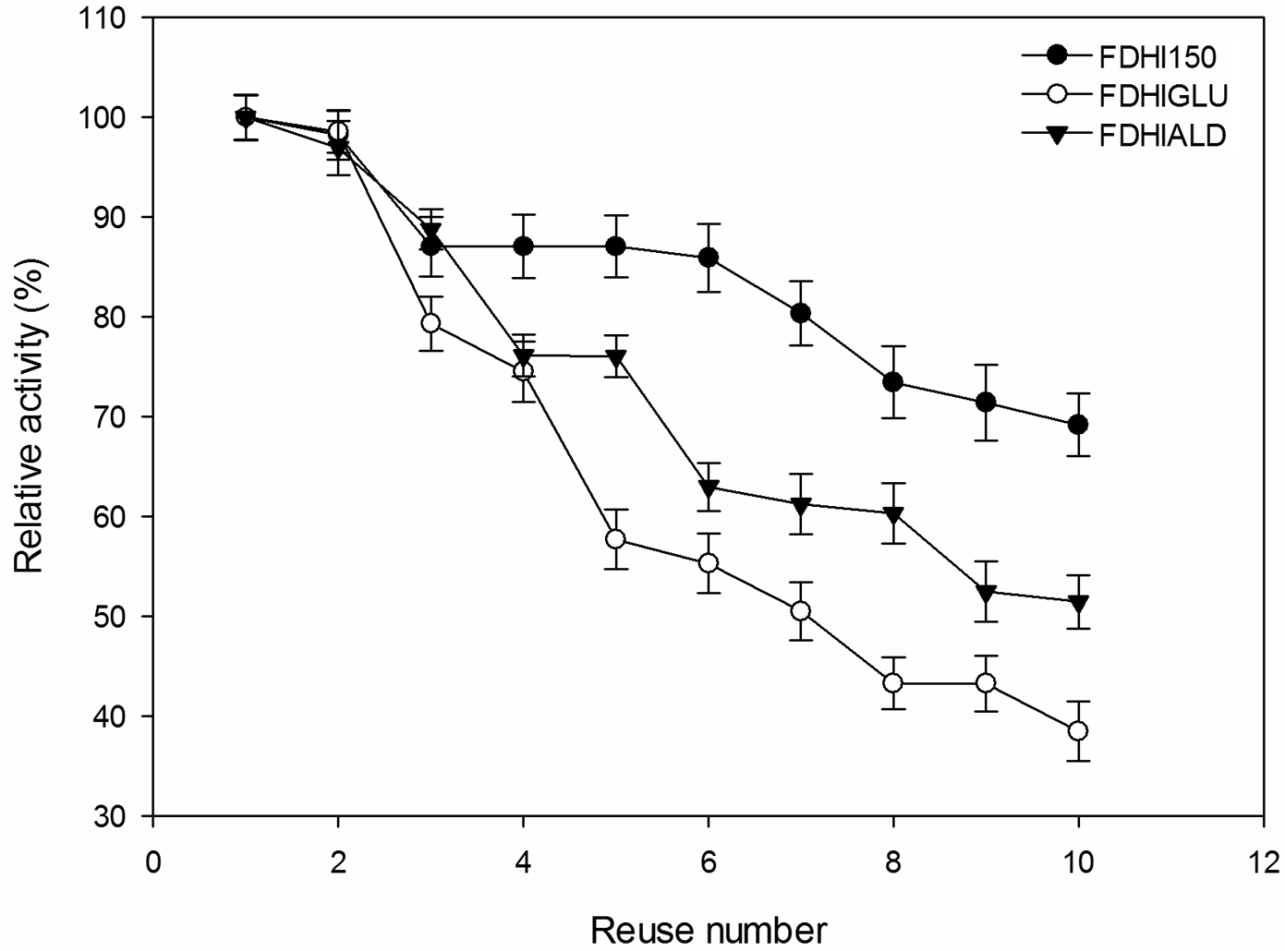
The reusability of immobilized FDHs.

It is an important feature to reuse a biocatalyst for many cycles without loss of initial activity. In this study, the operational stability of immobilized FDHs was tested in the batch type reactor for 10 reuses (Figure 6). The immobilized FDHs nearly protected their initial activities after 2 reuses. The remaining activities of FDHI150, FDHIGLU and FDHIALD were 69, 38 and 51%, respectively after 10 reuses. Gao et al. [31] reported that mutant FDH immobilized onto PD-IONPs protected 60% of its initial activity after 17 cycles. Kim et al. [17] determined that *C. boidinii* FDH immobilized as cross-linked enzyme aggregates prepared with dextrane polyaldehyde and glutaraldehyde, retained 96 and 89% of their initial activities, respectively after 10 reuses.

## Conclusion

In this study, the covalent immobilization of *C. methylica* FDH onto Immobead 150 support and modified Immobead 150 supports were investigated. A higher immobilization yield was obtained when tthe Immobead 150 support functionalized with aldehyde groups was used as support. Of the tested FDH preparations, FDHIALD showed highest catalytic efficiency and stability than the free FDH, FDHI150 and FDHIGLU. FDHI150, FDHIGLU and FDHIALD retained 69, 38 and 51% of their initial activities, respectively after 10 reuses. In conclusion, Immobead 150 support functionalized with aldehyde groups may be a potential candidate for the immobilization of enzymes and the immobilized FDHs, especially FDHIALD, is a robust biocatalyst and it may be used in the combination with other dehydrogenases to regenerate NADH.

## Experimental

Nicotinamide adenine dinucleotide hydrate (NAD^+^) was purchased from Acros Organics (New Jersey, USA). Sodium formate, Immobead 150 (Polyacrylic matrix, particle size 250 μm, oxirane content ≥200 μmol/g dry support), ethylenediamine (EDA), glutaraldehyde and sodium metaperiodate were obtained from Sigma-Aldrich (St. Louis, MO, USA). All other chemicals used in this study were of analytical grade and used without further purification.

### Purification of *C. methylica* FDH

The purification of FDH was performed according to Demir et al. [32]. Briefly, 7 g of wet *E. coli* BL21 (DE3) cell paste containing the expressed FDH protein was suspended in 10 mL buffer solution (20 mM Tris–HCl, pH 7.8, 0.5 M NaCl, 5 mM imidazole) at 4 °C. Then, the cells were disrupted by sonication and the sonicated cells were harvested by centrifugation (28000 × *g*, 30 min) at 4 °C. The cell pellet was resuspended in an ice-cold buffer (20 mM NaH_2_PO_4_, 0.5 M NaCl, 30 mM imidazole, pH 7.4). The resuspended cells were further lysed by adding lysozyme. Then the lysate was filtered through a 0.45 μm filter. The filtered samples were loaded to a His-trap column after equilibration with 5 mL of the ice-cold buffer. Then the column was washed with 5 mL of the same buffer. FDH was eluted with a series of elution buffers: 3 mL of elution buffer (20 mM phosphate buffer, 0.5 M NaCl with 100 mM imidazole pH 7.4), 5 mL of elution buffer (20 mM phosphate buffer, 0.5 M NaCl with 0.2 M imidazole pH 7.4), and finally 3 mL of elution buffer (20 mM phosphate buffer, 0.5 M NaCl with 0.4 M imidazole pH 7.4). The collected fractions were analyzed on SDS-PAGE.

### Preparation of modified supports

The modification of Immobead 150 support with EDA and glutaraldehyde was performed according to Yildirim et al. [33]. One gram of Immobead 150 support was treated with 10 mL of EDA solution (1 M in water, pH 10) for 12 h with mild stirring at room temperature. Then, the obtained supports were washed with distilled water and then dried at room temperature. One gram of EDA treated support was mixed with 25 mL phosphate buffer (50 mM, pH 7.0) containing 2.5% glutaraldehyde (w/v). After gently 2 h stirring, the supports were washed with distilled water and then dried at room temperature.

One gram of Immobead 150 support was treated with 10 mL of 1 M acetic acid solution for 12 h with mild stirring at room temperature. Then, the obtained supports were washed with distilled water and then dried at room temperature. One gram of the support was added onto 25 mL of sodium meta periodate solution. After 2 h stirring time the supports were washed with distilled water and then dried at room temperature.

### Immobilization of FDH

The covalent immobilization of FDH onto Immobead 150 support was performed according to Alagöz et al. [34]. One gram of Immobead 150 support was mixed with 9.0 mL of FDH solution containing 1.0 mg/mL protein in 1.0 M, pH 7.0 phosphate buffer. The mixture was gently shaken at 25 °C in a water bath during 24 h immobilization time. The immobilized FDH preparations were filtrated to collect them and washed with distilled water.

The covalent immobilization of FDH onto Immobead 150 via a glutaraldehyde spacer arm was performed according to Yildirim et al. [33] with slight modification. One gram of the modified support was treated with 9.0 mL of FDH solution containing 1.0 mg/mL protein in 50 mM, pH 7.0 phosphate buffer. The immobilization was allowed to continue in a water bath at 5 °C for 4 h with slow shaking. Then, the immobilized FDH preparations were filtrated to collect them and washed with distilled water.

The covalent immobilization of FDH onto Immobead 150 functionalized with aldehyde groups was carried out by adding 9.0 mL of FDH solution containing 1.0 mg/mL protein in 50 mM, pH 6.0 citrate buffer onto 1 g of the support. The immobilization was allowed to continue in a water bath at 5 °C for 4 h with slow shaking. Then, the immobilized FDH preparations were filtrated to collect them and washed with distilled water.

The protein contents of filtrates were checked by measuring their absorbance values at 280 nm and the washing procedure was continued until no absorbance were detected in the filtrates. After that, the immobilized FDH preparations were stored at 5 °C until use. The amounts of immobilized protein onto the supports were determined using a Bradford protein assay [35].

### FDH assay

The FDH activity was measured spectrophotometrically at 340 nm according to Özgün et al. [36]. Five milligrams of immobilized FDH or 50 µL of free FDH (5.4 mg protein/mL), 2.6 mL of phosphate buffer (0.1 M, pH 7.0) and 0.5 mL of 0.1 M sodium formate solution (0.1 M in pH 7.0 phosphate buffer) were mixed in a test tube. The reaction was started by the addition of 0.1 mL NAD^+^ solution (10 mM in water) at 25 °C in a water bath. After 10 min reaction time, an aliquot of 3 mL was taken from the reaction mixture and its absorbance was measured at 340 nm. The same procedure was applied to a blank tube containing no free or immobilized FDH sample. One unit of FDH activity was defined as the amount of enzyme produced 1.0 µmol of CO_2_ from formate in the presence of NAD^+^ under the assay conditions.

### Characterization of FDH

The effect of pH on the activities of free and immobilized FDHs was investigated at different pHs ranging from 5.0 to 8.0 at 35 °C. The optimal temperatures of free and immobilized FDH preparations were determined in a temperature range of 25–50 °C at pH 7.0.

The thermal stability of free and immobilized FDH preparations was tested by incubating the preparations at 35 and 50 °C and measuring the activities of the samples in certain time intervals.

### Operational stability of immobilized FDH

The operational stability of the immobilized FDHs was investigated in a batch type column reactor. The immobilized FDH preparation (0.1 g of each) was loaded to the reactor and 2.6 mL of phosphate buffer (0.1 M, pH 7.0) and 0.5 mL of 0.2 M sodium formate solution (0.1 M in pH 7.0 phosphate buffer) were added. The reaction was started by the addition of 0.1 mL NAD^+^ solution (10 mM in water) at 25 °C in a water bath. The reaction mixture was separated from the immobilized FDH and its absorbance was measured at 340 nm. For the next cycle, the immobilized FDH was rinsed with the phosphate buffer (5 mL) and the freshly prepared reaction mixture was added onto it.
